# Image Processing-Based Recognition of Wall Defects Using Machine Learning Approaches and Steerable Filters

**DOI:** 10.1155/2018/7913952

**Published:** 2018-11-15

**Authors:** Nhat-Duc Hoang

**Affiliations:** Lecturer, Faculty of Civil Engineering, Institute of Research and Development, Duy Tan University, P809-03 Quang Trung, Da Nang, Vietnam

## Abstract

Detection of defects including cracks and spalls on wall surface in high-rise buildings is a crucial task of buildings' maintenance. If left undetected and untreated, these defects can significantly affect the structural integrity and the aesthetic aspect of buildings. Timely and cost-effective methods of building condition survey are of practicing need for the building owners and maintenance agencies to replace the time- and labor-consuming approach of manual survey. This study constructs an image processing approach for periodically evaluating the condition of wall structures. Image processing algorithms of steerable filters and projection integrals are employed to extract useful features from digital images. The newly developed model relies on the Support vector machine and least squares support vector machine to generalize the classification boundaries that categorize conditions of wall into five labels: longitudinal crack, transverse crack, diagonal crack, spall damage, and intact wall. A data set consisting of 500 image samples has been collected to train and test the machine learning based classifiers. Experimental results point out that the proposed model that combines the image processing and machine learning algorithms can achieve a good classification performance with a classification accuracy rate = 85.33%. Therefore, the newly developed method can be a promising alternative to assist maintenance agencies in periodic building surveys.

## 1. Introduction

During the construction and maintenance of high-rise buildings, it is very crucial to attain good surface quality of structures due to safety and esthetics aspects. Because of the combined effects of aging, weather conditions, and human activities, the condition of building structures deteriorates over time [1]. If left untreated, damages such as cracks and spalls obviously cause inconvenience for the building's occupants, deteriorate the structural integrity, and lead to a significant reduction of the value of the poorly maintained asset. Therefore, identifying defective areas that appear on surface structure is one of the main tasks in periodic survey buildings.

In high-rise buildings, the components that have large surface areas typically include walls (both concrete walls and brick walls covered by mortar) and slabs. The assessment of concrete slabs is usually performed during the construction phase. It is because during the operation phase, the surface of slab structure is concealed by floor coverings such as ceramic or stone tiles. Therefore, this study focuses on the visual assessment of wall structures.

In Vietnam, periodic surveys on building condition are usually performed by visual assessment of human inspectors. This fact is also common in other countries because visual changes in structures can directly point out the potential problems of building structures [2]. For instances, cracks can be indicators of structural problems including building settlement and degradation of building materials; particularly for concrete walls, spalls can be caused by the corrosion of embedded reinforcement bars [3–5].

In the current practice of building condition assessment, the damages on the surface of building structures are usually inspected by qualified technicians. These technicians often utilize contact-type equipment including profilometer and measuring tape for identifying the defective areas [6]. Although the manual procedure can help to obtain accurate condition of the structure, it also has several disadvantages. First, the surveying process is strongly affected by the knowledge, experience, and subjective judgment of human inspectors; therefore, this issue can lead to inconsistency of the assessment outcome. Second, the process of visual assessment, measurement, data processing, and report can be very time consuming especially for high-rise buildings with large surface areas needed to be inspected periodically.

Accordingly, it is immensely beneficial for the building owners and maintenance agencies if the manual inspection process can be replaced by a more productive and consistent method of surveying [7]. Among automated methods for building condition evaluation, machine vision based approaches are widely employed due to their ease of access to equipment, their fast computing processes, and the rapid advancements of image processing techniques [8–14].

Hutchinson and Chen [15] presented a statistical-based method for evaluating concrete damage including cracks and spalls and relied on a Bayesian method for recognizing cracks automatically from images. Chen et al. [16] employed the first derivative of a Gaussian filter to analyze multitemporal images for measuring cracks. Zhu [17] put forward an intelligent method using three circular filters to detect air pockets appearing on the surfaces of concrete.

The level set method and morphological algorithms for image processing have been used by Chen and Hutchinson [18] to identify and analyze cracks in laboratory environment. Lee et al. [19] proposed a model that integrates various image processing operations (brightness adjustment, binarisation, and shape analysis) to facilitate the accuracy of crack detection; in addition, a neural network-based model was implemented to classify crack patterns of cracks. Valença et al. [20] introduced a method based on multispectral image analysis to evaluate and delineate defective areas.

An image processing-based approach for detecting bugholes on concrete surface has been established by Liu and Yang [21]; the employed techniques are contrast enhancement and Otsu thresholding. Kim et al. [22] compared different image binarisation algorithms for identifying cracks in concrete structures. Hoang [23] employed the Otsu method and a gray intensity modification approach for binarizing images and isolating cracks.

Silva and Lucena [24] demonstrated the capability of deep learning approach for concrete crack detection. Dorafshan et al. [25] has recently compared the performances of deep convolutional neural networks and edge detection methods for the task of recognizing concrete cracks. The notable advantage of deep learning approaches is that their feature extraction operators are autonomously constructed during the model training phase [26]. However, methods based on deep learning often necessitate a considerable amount of training samples and require a large computational cost.

As can be seen from the existing literature, most of the previous works have dedicated in constructing models for the classification of crack and noncrack conditions. Few studies have constructed an integrated image processing model for detect cracks and spalls. The objective of the current study is to combine image processing techniques and advanced machine learning algorithms into an integrated model that is capable of detecting and categorizing the defective areas on wall structures. By using an intelligent model that can recognize and categorize types of cracks and spalling areas simultaneously, the task of periodic building condition survey can be executed in a more effective manner.

The feature extraction phase of the new model relies on image processing algorithms of steerable filters and projection integrals. It is because these two algorithms of image processing have demonstrated their usefulness in recognizing defects in pavements [27, 28]. Based on the extracted features, machine learning algorithms including support vector machine (SVM) and least squares support vector machine (LSSVM) are employed to classify input images into five labels: longitudinal cracks, transverse cracks, diagonal cracks, spalls, and intact walls. The reason for the selection of these machine learning approaches is that their outstanding performances in classification tasks have been reported in the literature [29–33].

Based on the aforementioned features, the main contributions of the newly constructed model can be summarized as follows:

- Multiple types of cracks (longitudinal cracks, transverse cracks, diagonal cracks) existing in wall structures can be detected and categorized in an integrated model. This can be considered to be a significant improvement since most of the existing models can only produce the prediction outcome of crack or noncrack conditions [13, 23–25].
- Although projection integrals have been utilized in structural defect classification [27, 28], diagonal projection integrals which have been rarely exploited in concrete surface crack categorization are employed in this study to specifically deal with diagonal cracks.
- Cracks and spall damages can be recognized simultaneously in an integrated model which has also rarely been achieved in the current literature.
- Neural networks have been extensively used in concrete crack categorization [19, 34, 35]. However, the applications of SVM and LSSVM in this task are still limited and a comparative work needs to be performed to evaluate the potential of these two advance machine learning algorithms in dealing with the problem of wall defect recognition.

The rest of the paper is organized as follows: the research methodology is reviewed briefly in the next section, followed by the description of the newly constructed automatic approach for wall defect detection; the fourth section reports experimental results of this study, followed by the conclusion in the final section.

## 2. Research Methodology

### 2.1. Image Processing Approaches

#### 2.1.1. Steerable Filter (SF)

SF [36] is an orientation-selective convolution kernel used widely used for feature extraction. In image processing field, oriented filters are often employed in various vision and image processing tasks including edge detection and texture analysis [37]. Since cracks and spalls on wall surface have distinctive edges and patterns; the utilization of SF can be helpful to recognize these defects.

SF is based on the computation of directional derivatives of Gaussians; accordingly, these filters can be used to construct local orientation maps of a digital image [37]. SF is essentially a linear combination of Gaussian second derivatives. For an image *I *(*x*,*y*), a 2-*D* Gaussian at a certain pixel is computed in the following formula [38]:

(1) $$\begin{array}{c} G\left(x,y,r\right)=\frac{1}{\sqrt{2\pi r}}\exp\frac{-\left(x^{2}+y^{2}\right)}{2r^{2}}, \end{array}$$

where *r* is a free parameter which denotes the Gaussian function variance.

The expression of the SF formulation with an orientation of *θ* is given as follows:

(2) $$\begin{array}{c} F\left(x,y,r,\theta \right)=G_{xx}{\;\cos}^{2}\left(\theta \right)+2G_{xy}\cos\left(\theta \right)\sin\left(\theta \right)+G_{yy}{\;\sin}^{2}\left(\theta \right), \end{array}$$

where *G*_*xx*_, *G*_*xy*_, and *G*_*yy*_ denote the Gaussian second derivatives and their expression are given in the following equations:

(3) $$\begin{array}{c} G_{xx}\left(x,y,r\right)=\frac{\left(x^{2}-r^{2}\right)\exp\left(-\left(x^{2}+y^{2}\right)/2r^{2}\right)}{\sqrt{2\pi }r^{5}},\\ G_{yy}\left(x,y,r\right)=\frac{\left(y^{2}-r^{2}\right)\exp\left(-\left(x^{2}+y^{2}\right)/2r^{2}\right)}{\sqrt{2\pi }r^{5}},\\ G_{xy}\left(x,y,r\right)=G_{xy}\left(x,y,r\right)=\frac{xy\exp\left(-\left(x^{2}+y^{2}\right)/2r^{2}\right)}{\sqrt{2\pi }r^{5}}. \end{array}$$

**Figure 1:**
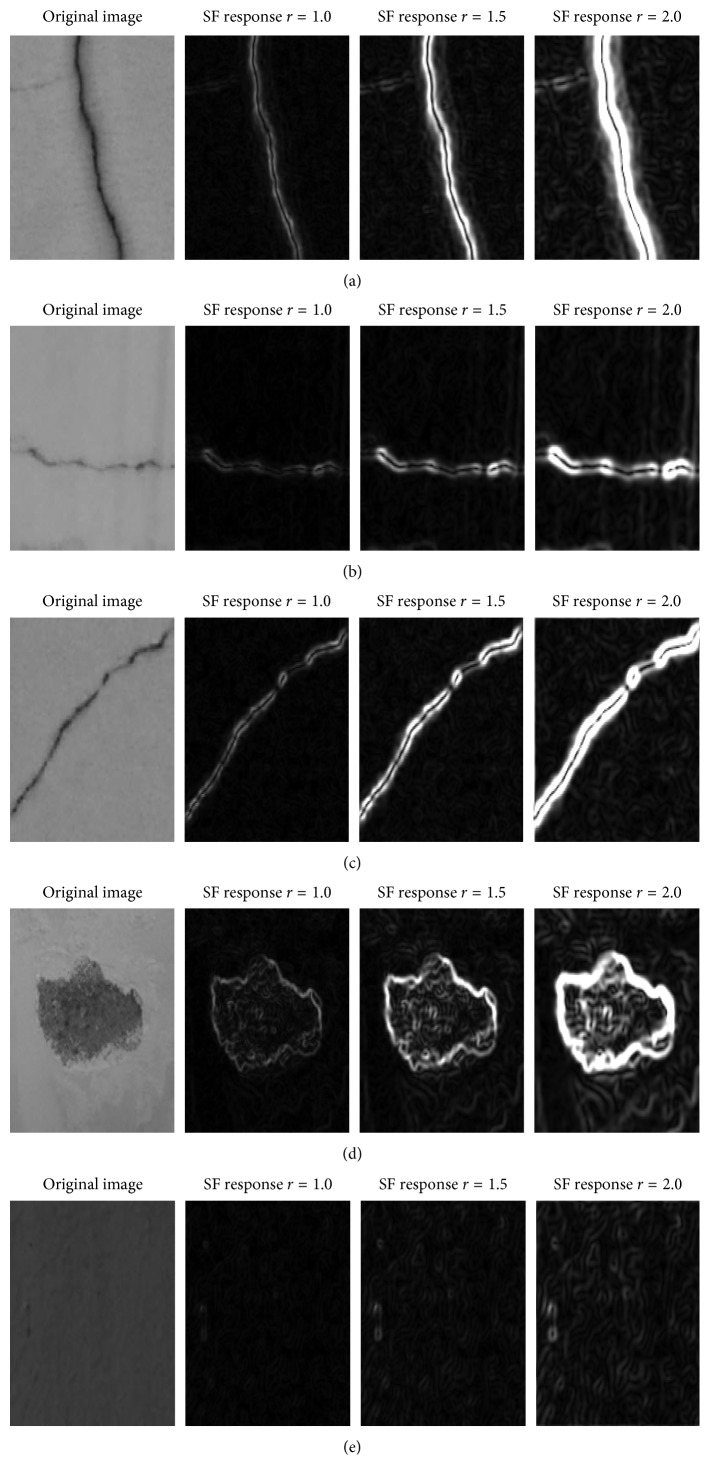
SF responses: (a) longitudinal crack, (b) transverse crack, (c) diagonal crack, (d) spall damage, and (e) intact wall.

Notably, if the value of the parameter *r* which is the variance of the Gaussian function variance is fixed, the final response map of an image is obtained by combining the outcomes of individual SFs with different values of *θ*. In this study, the values of *θ* vary from 0° to 360° with an interval of 30°. The responses of SFs of images containing defects are demonstrated in Figure 1 with different values of the Gaussian function variance.

In addition, the final response map created by SFs for an image *I* is calculated using the equation below:

(4) $$\begin{array}{c} R\left(x,y\right)=F\left(x,y,\sigma ,\theta \right)\;*\;I\left(x,y\right), \end{array}$$

where “*∗*” is the symbol of the convolution operator.

#### 2.1.2. Projection Integral (PI)

PI is a widely used method for image analysis. This approach is particularly useful for shape and texture categorization and has been extensively employed for face and facial recognition [39, 40]. In the field of civil engineering, this image analysis method has been successfully applied in pavement crack classification tasks [27, 38, 41] as well as pavement pothole recognition [28].

**Figure 2:**
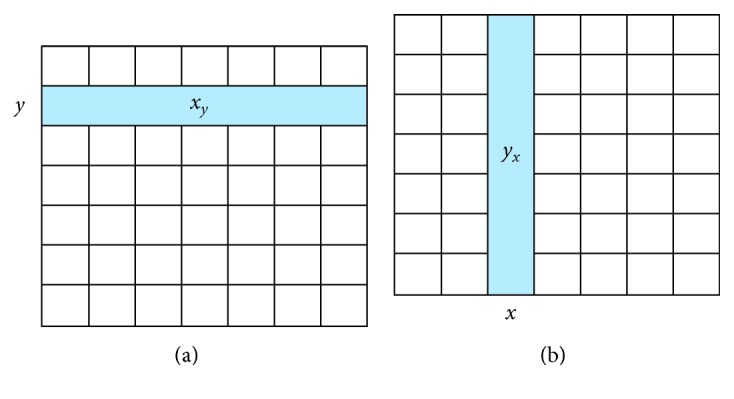
Illustration of PIs of an image: (a) HPI and (b) VPI.

The two PIs along the horizontal and vertical axes of an image are denoted as horizontal PI (HPI) and vertical PI (VPI). These two PIs are computed according to the following equations:

(5) $$\begin{array}{c} \text{HPI}\left(y\right)=\underset{i\in x_{y}}{\sum }I\left(i,y\right),\\ \text{VPI}\left(x\right)=\underset{j\in y_{x}}{\sum }I\left(x,j\right), \end{array}$$

where *x*_*y*_ and *y*_*x*_ are the set of horizontal pixels at the location *y* and the set of vertical pixels at the location *x*, respectively (Figure 2).

**Figure 3:**
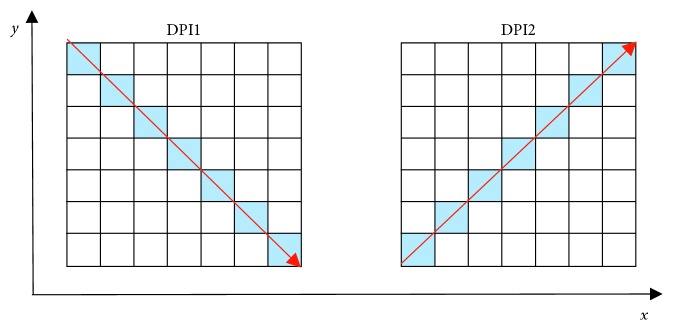
Illustration of DPIs of an image.

Since HPI and VPI are incapable of detecting diagonal cracks [42], the two diagonal PIs (DPIs) of an image are employed. The directions of the two DPs, denoted as DPI1 and DPI2, are illustrated in Figure 3. The two DPIs are calculated as follows:

(6) $$\begin{array}{c} \text{DPI}1\left(x,y\right)=\underset{x,y\in D1}{\sum }I\left(x,y\right),\\ \text{DPI}2\left(x,y\right)=\underset{x,y\in D2}{\sum }I\left(x,y\right), \end{array}$$

where *D*1 and *D*2 are the set of pixels along the two diagonal directions of an image (as illustrated in Figure 3).

**Figure 4:**
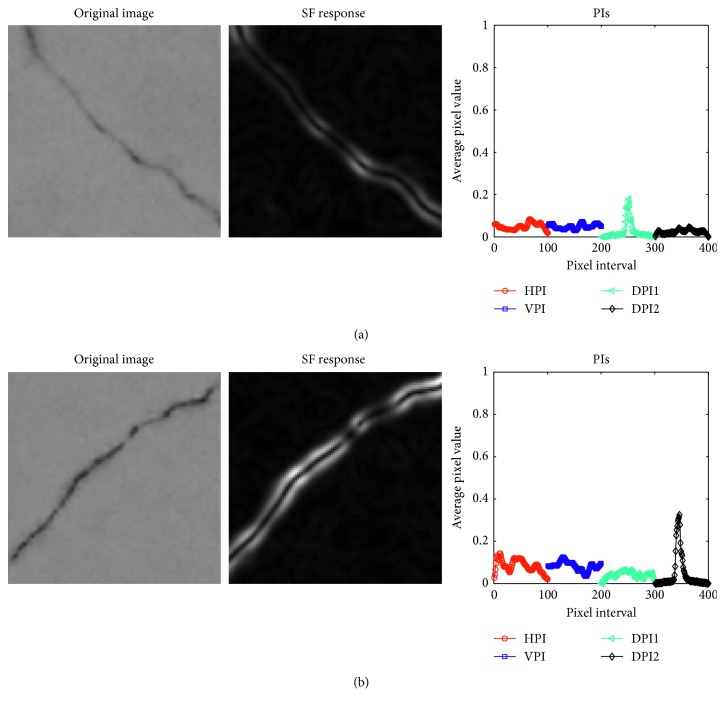
PIs of diagonal cracks.

**Figure 5:**
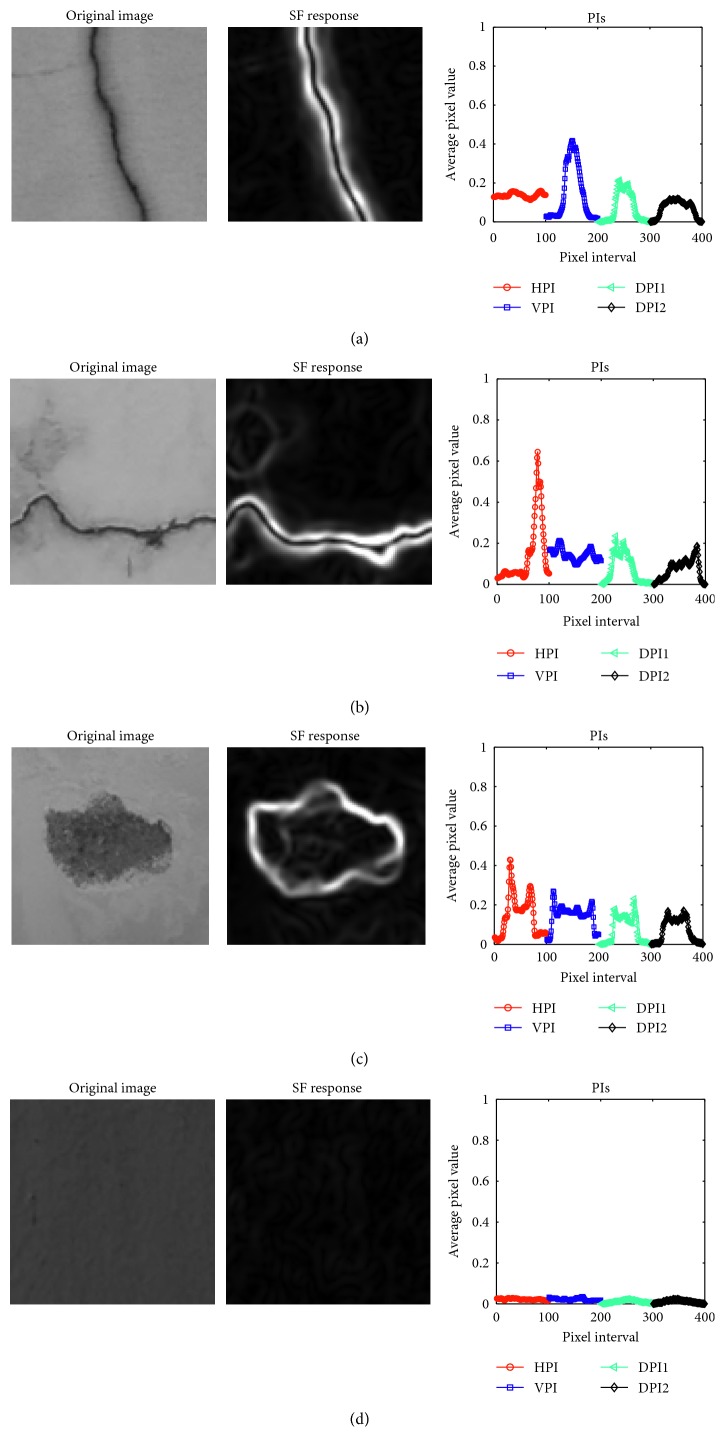
PIs of image samples: (a) longitudinal crack, (b) transverse crack, (c) spall damage, and (d) intact wall.

As illustrated in Figure 4, images containing diagonal cracks have PIs in which there are exceptionally high intensities in the two DPIs. In addition, as can be shown in Figure 5, HPI and VPI have the strongest responses of PI in images having longitudinal and transverse cracks. On the other hand, an image containing a spall damage results in PIs which do not have a significant peak of signal and the average value of its SF response is higher than that of an image without defective areas.

### 2.2. Support Vector Machine and Least Squares Support Vector Machine

Support vector machine (SVM) is a machine learning based classifier which is established on the basis of the statistical learning theory [43]. The aim of this learning algorithm is to find a predictive function based on the collected data set. The standard version of SVM is designed to cope with binary or two-class pattern-recognition problems. Through the model construction phase, SVM constructs a hyperplane to classify data points so that the distance from it to the nearest data sample of each class label is maximized [44].

**Figure 6:**
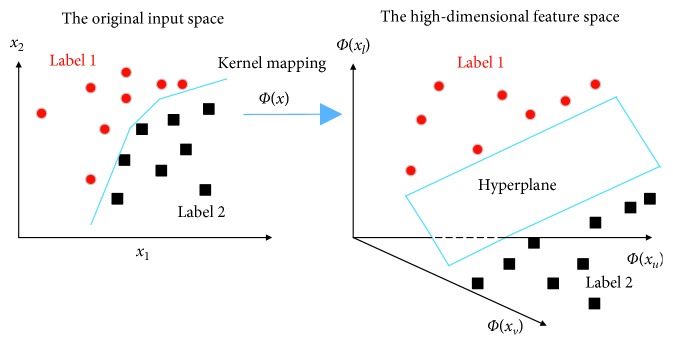
The learning phase of SVM.

Moreover, this algorithm relies on the kernel trick to better deal with nonlinearly separable cases. Using the kernel trick, the data points are mapped from an original input space to a high-dimensional feature space so that linear separability is easier to achieve (Figure 6). Superior classification performance of SVM has been widely reported in a large number of previous studies [45–49].

Given a set of training data points {*x*_*k*_, *y*_*k*_}_*k*=1_^*N*^ with input data *x*_*k*_ ∈ *R* ^*n*^ and a set of class labels *y*_*k*_ ∈ {−1, +1}, the model construction phase of SVM is equivalent to solving the following optimization problem:

(7) $$\begin{array}{c} \text{minimize}J_{p}\left(w,e\right)=\frac{1}{2}w^{T}w+c\frac{1}{2}\overset{N}{\underset{k=1}{\sum }}e_{k}^{2},\\ \text{subject to}y_{k}\left(w^{T}\varphi \left(x_{k}\right)+b\right)\geq 1-e_{k},k=1,...,N,e_{k}\geq 0, \end{array}$$

where *w* ∈ *R* ^*n*^ and *b* ∈ *R* denote the model parameters, *e*_*k*_ > 0 represents a slack variable, *c* denotes a penalty constant which determines severity of learning error, and *φ*(*x*) denotes a nonlinear mapping from the input space to the feature space.

One of the advantages of SVM is that it does not require expressing the mapping function *φ*(*x*) explicitly. Due to the concept of kernel trick, the model identification only necessitates the computation of the kernel function *K*(.) which is the dot product of *φ*(*x*). The kernel function is shown below:

(8) $$\begin{array}{c} K\left(x_{k},x_{l}\right)=\varphi {\left(x_{k}\right)}^{T}\varphi \left(x_{l}\right). \end{array}$$

Radial basis function (RBF) is often selected to be used in SVM [30]; its formula is given as follows:

(9) $$\begin{array}{c} K\left(x_{k},x_{l}\right)=\exp\left(-\frac{{\left\|x_{k}-x_{l}\right\|}^{2}}{2\sigma^{2}}\right), \end{array}$$

where *σ* denotes the kernel function parameter.

To solve the aforementioned constrained optimization, the Lagrangian is given as follows:

(10) $$\begin{array}{c} L\left(w,b,e;\alpha ;v\right)=J_{p}\left(w,e\right)-\overset{N}{\underset{k=1}{\sum }}\alpha_{k}\left\{y_{k}\left(w^{T}\varphi \left(x_{k}\right)+b\right)-1+e_{k}\right\}-\overset{N}{\underset{k=1}{\sum }}v_{k}e_{k}, \end{array}$$

where *α*_*k*_ ≥ 0, *v*_*k*_ ≥ 0 denote Lagrange multipliers for *k* = 1, 2,… , *N*.

Accordingly, the conditions for optimality are stated as follows:

(11) $$\begin{array}{c} \left\{\begin{array}{c} \frac{\partial L}{\partial w}=0⟶w=\overset{N}{\underset{k=1}{\sum }}\alpha_{k}y_{k}\varphi \left(x_{k}\right),\\ \frac{\partial L}{\partial b}=0⟶\overset{N}{\underset{k=1}{\sum }}\alpha_{k}y_{k}=0,\\ \frac{\partial L}{\partial e_{k}}=0⟶0\leq \alpha_{k}\leq c,k=1,...,N. \end{array}\right. \end{array}$$

Based on the equations found by the conditions for optimality, it is able to attain the following dual quadratic programming problem:

(12) $$\begin{array}{c} \underset{\alpha }{\max}J_{D}\left(\alpha \right)=-\frac{1}{2}\overset{N}{\underset{k,l=1}{\sum }}y_{k}y_{l}\varphi {\left(x_{k}\right)}^{T}\varphi \left(x_{l}\right)\alpha_{k}\alpha_{l}+\overset{N}{\underset{k=1}{\sum }}\alpha_{k},\\ \text{Subject to}\overset{N}{\underset{k=1}{\sum }}\alpha_{k}y_{k}=0,0\leq \alpha_{k}\leq c,\;k=1,\ldots ,N, \end{array}$$

In addition, the kernel function is applied in the following manner:

(13) $$\begin{array}{c} \omega =y_{k}y_{l}\varphi {\left(x_{k}\right)}^{T}\varphi \left(x_{l}\right)=y_{k}y_{l}K\left(x_{k},x_{l}\right). \end{array}$$

Finally, the classification model based on SVM can be derived as follows:

(14) $$\begin{array}{c} y\left(x\right)=\mathrm{sign}\left(\overset{SV}{\underset{k=1}{\sum }}\alpha_{k}y_{k}K\left(x_{k},x_{l}\right)+b\right), \end{array}$$

where SV is the number of support vectors which are training data points that have *α*_*k*_ > 0.

Least squares support vector machine (LSSVM) [50] is a least square version of the original SVM. Instead of solving a quadratic programming problem required by SVM, the model construction phase of LSSVM is equivalent to solving a system of linear equation. Therefore, the computational expense of LSSVM can be much lower than that of the standard SVM.

To construct a LSSVM based classifier, it is needed to solve the following minimization problem:

(15) $$\begin{array}{c} \text{minimize}J_{p}\left(w,e\right)=\frac{1}{2}w^{T}w+\gamma \frac{1}{2}\overset{N}{\underset{k=1}{\sum }}e_{k}^{2},\\ \text{subject to}y_{k}\left(w^{T}\varphi \left(x_{k}\right)+b\right)=1-e_{k},k=1,...,N, \end{array}$$

where *w* ∈ *R* ^*n*^ and *b* ∈ *R* are also the model parameters; *e*_*k*_ ∈ *R* denote error variables; *γ* > 0 is called a regularization constant.

Subsequently, the Lagrangian is applied in the following way:

(16) $$\begin{array}{c} L\left(w,b,e;\alpha \right)=J_{p}\left(w,e\right)-\overset{N}{\underset{k=1}{\sum }}\alpha_{k}\left\{y_{k}\left(w^{T}\varphi \left(x_{k}\right)+b\right)-1+e_{k}\right\}, \end{array}$$

where *α*_*k*_ is the *k* ^th^ Lagrange multiplier; *φ*(*x*_*k*_) represents a nonlinear mapping function.

Relied on the KKT conditions for optimality, it is able to convert the aforementioned constrained optimization problem to a linear system [51]. The classifier based on LSSVM is compactly expressed as follows:

(17) $$\begin{array}{c} y\left(x\right)=\mathrm{sign}\left(\overset{N}{\underset{k=1}{\sum }}\alpha_{k}y_{i}K\left(x_{k},x_{l}\right)+b\right), \end{array}$$

where *α*_*k*_ and *b* are found by solving a linear system. Similar to the standard SVM, *K*(*x*_*k*_, *x*_*l*_) denotes the kernel function.

## 3. Image Sample Collection

Because the machine learning algorithms of SVM and LSSVM are supervised learning approach, a set of wall images with the corresponding ground truth labels must be prepared in advance of the model construction and classification phases. To establish the required data set, images of walls have been collected during field surveys at high-rise buildings in Da Nang city (Vietnam).

**Figure 7:**
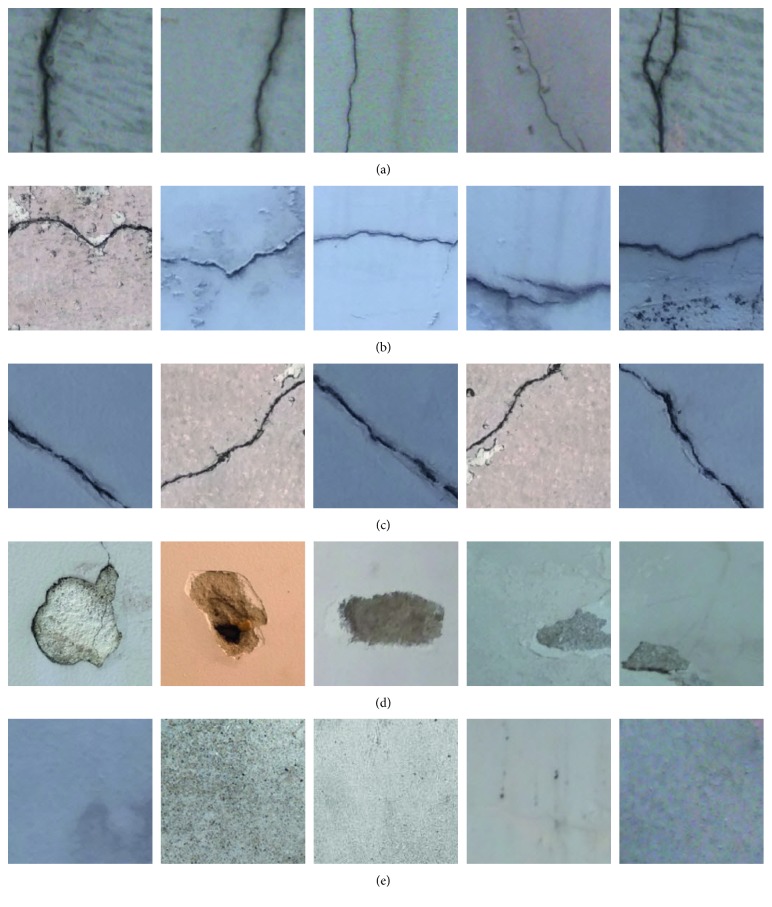
The collected image samples: (a) longitudinal crack, (b) transverse crack, (c) diagonal crack, (d) spall damage, and (e) intact wall.

To ease the computational process, the size of each image sample is fixed to be 200 × 200 pixels. Moreover, there are five classes of wall condition, namely, longitudinal cracks (LC), transverse crack (TC), diagonal cracks (DC), spall damage (SD), and intact wall (IW). The number of image samples in each class is 100. Thus, the collected image data set contains 500 samples and is illustrated in Figure 7. It is also noted that all the image samples have been preprocessed by the median filter with a window size of 5 × 5 pixels. This preprocessing step aims at suppressing the noise existing in the collected digital image [52]. In addition, the data set is divided into two folders that contain the training set of images (90%) and the testing set of images (10%). The first set is used in the phase of model construction and the second set is utilized to verify the generalization capability of the wall defect classification model.

## 4. The Proposed Hybrid Approach of Image Processing and Machine Learning for Detection of Wall Defects

**Figure 8:**
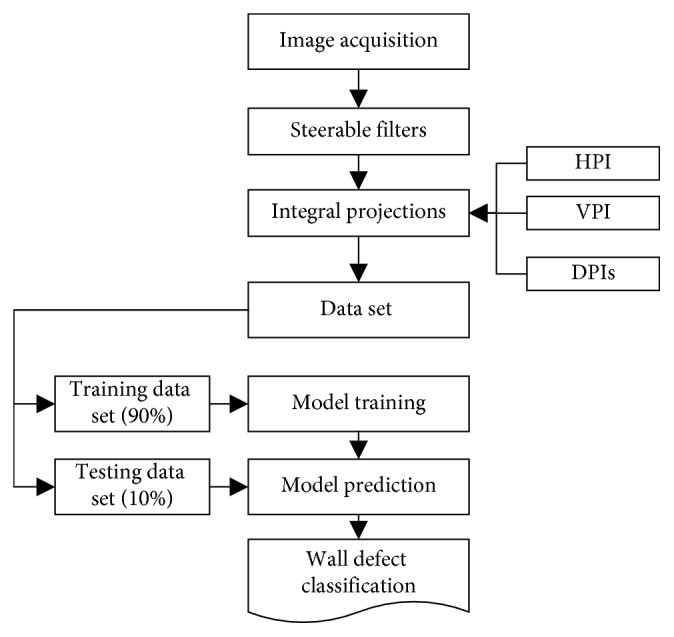
The proposed model structure.

This section describes the proposed model used for automatic classification of wall defects. The overall model structure is illustrated in Figure 8. The model can be divided into two separated modules:

- Feature extraction phase that employs the image processing methods of SFs and IPs
- Machine learning based classification phase that relies on the SVM and LSSVM algorithms

In the first step of feature extraction, SFs are employed to compute a salient defect map from a digital image. The minimum and maximum angles of SFs are 0° and 360°, respectively. The parameter *r* of SFs is selected from a set of [1.0, 1.5, 2.0, 2.5, 3.0]. The value of *r* which results in the highest accuracy for the training data set is selected as the optimal one. Based on the map generated by SFs, PIs including HPI, VPI, DPI1, and DPI2 are then computed to characterize the texture of the captured images. As earlier mentioned, each image of the data set has the size of 200 × 200 pixels. Thus, each PI contains 200 sampled points and the total number of features to be analyzed by the machine learning algorithm is 200 × 4 = 800. Herein, 4 is the number of PIs.

Obviously, the original PIs can be very rough and have many peaks and valleys due to local fluctuations of the gray intensity of an image. Moreover, the large number of input features can create difficulty for the machine learning algorithms of SVM and LSSVM due to the curse of dimensionality [53]. Therefore, it is beneficial to smooth the original PIs by the use of moving average method [42]. In detail, the average value of *W*_PI_ consecutive values along the PIs is calculated to create smoothed PIs with fewer data points. For example, if *W*_PI_ = 10, then the total number of features in the contracted PIs is reduced from 800 to 80; if *W*_PI_ = 20, then the machine learning algorithms only have to deal with a data set having 40 features.

**Figure 9:**
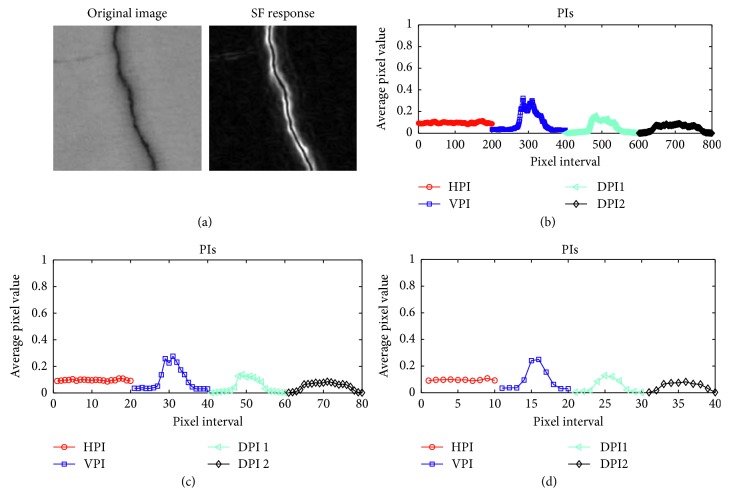
PIs of an image: (a) the original image, (b) the original PIs (*W*_PI_ = 1), (c) the smoothed PIs (*W*_PI_ = 10), and (d) the smoothed PIs (*W*_PI_ = 20).

The process of feature number reduction using moving average for an image is demonstrated in Figure 9. As can be seen from this example, the smoothed PIs even with the window size *W*_IP_ = 20 still preserve crucial features of rises and ebbs of the original PIs. Therefore, the value of *W*_IP_ = 20 is selected for the feature extraction step. Accordingly, the set of 40 features extracted from PIs is used as input pattern to categorize the four labels of wall defect (LC, TC, DC, and SD) and the label of intact wall (IW).

**Figure 10:**
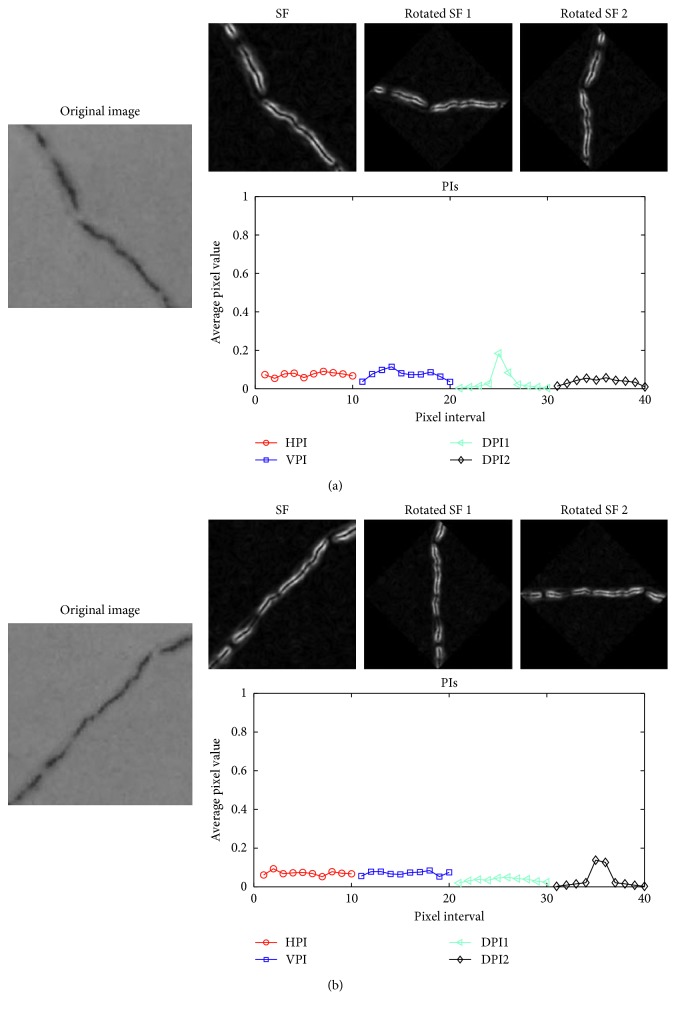
The process of computing DPIs: (a) a −45° diagonal crack and (b) a + 45° diagonal crack.

**Figure 11:**
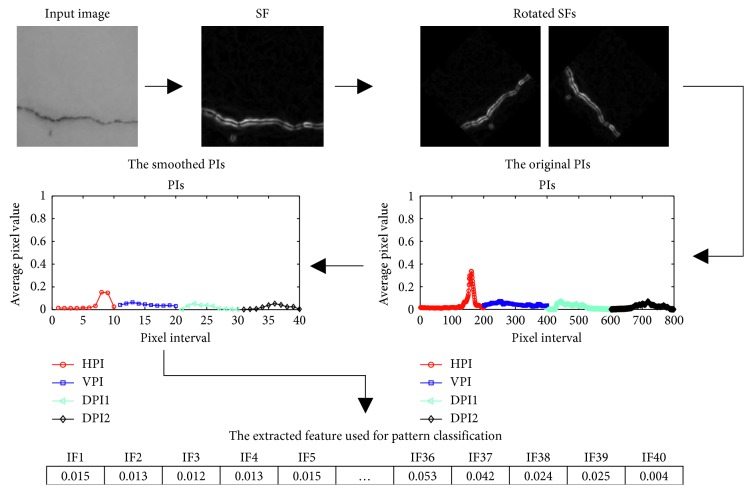
The whole feature extraction process.

Furthermore, to obtain the two DPIs of DPI1 and DPI2, the original map of the SF response has been rotated with the angles of +45 and −45 [42]. Accordingly, the two DPIs are derived from the computation of the HPIs of the two rotated SF maps. The process of computing DPIs of images containing diagonal cracks is demonstrated in Figure 10. In addition, the overall feature extraction module is depicted in Figure 11. Based on the 40 input features (IF) extracted from the PIs, the machine learning algorithms of SVM and LSSVM are employed to perform the model learning and classification of images in the second module of the model.

It is noted that the standard versions of SVM and LSSVM are designed two-class pattern recognition problems. Hence, the one-versus-one (OvO) strategy [54] has been used with SVM and LSSVM to make them capable of dealing with the five-class recognition tasks at hand. Previous works have confirmed the advantages of OvO strategy in coping with multiclass classification problems [53, 55, 56].

## 5. Experimental Results

Because the detection of wall defects is formulated as a five-class pattern recognition problem, classification accuracy rate (CAR) computed for each individual class and for all of the classes is employed. CAR for the class *i* is computed as follows:

(18) $$\begin{array}{c} \text{CA}{\text{R}}_{i}=\frac{R_{C}^{i}}{R_{A}^{i}}\times 100\left(\%\right), \end{array}$$

where *R*_*C*_^*i*^ and *R*_*A*_^*i*^ denote the number of data samples in class *i*th being correctly classified and the total number of data instances in this class, respectively. It is reminded that that there are five class labels in the data set: longitudinal crack (LC), transverse crack (TC), diagonal crack (DC), spall damage (SD), and intact wall (IW).

The overall classification accuracy rate (CAR) for all the five class labels is simply computed as follows:

(19) $$\begin{array}{c} \text{CA}{\text{R}}_{\text{Overall}}=\overset{5}{\underset{i=1}{\sum }}\frac{\text{CA}{\text{R}}_{i}}{5}. \end{array}$$

As stated earlier, the data set including 500 samples is employed to train and test the wall defect classification model. This data set is randomly separated into two subsets: data for model training (90%) and data for testing (10%). Because a single run of model training and testing may not help to reveal the true performance of a classifier, this study performs a repeated subsampling process that includes 20 times of model training and prediction. In each time of running, 10% of the data set is randomly extracted to form the testing data subset; the rest of the data set is reserved for model testing phase.

**Figure 12:**
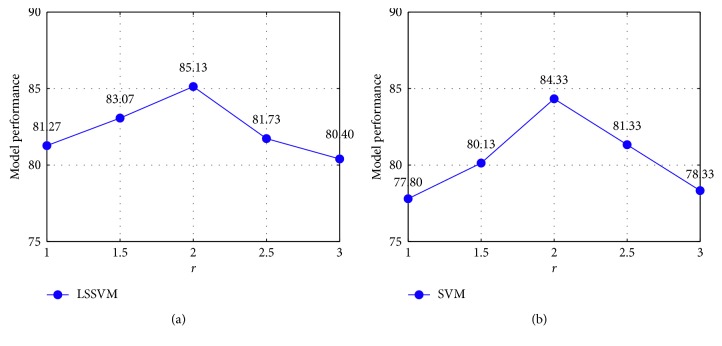
Model performance with different values of the parameter *r*: (a) LSSVM and (b) SVM.

As described in the formulation of the two machine learning algorithms of SVM and LSSVM, these two algorithms both require a proper setting of their tuning parameters. In the case of SVM, the tuning parameters are the penalty coefficient and the kernel function parameter. In the case of LSSVM, the regularization and the kernel function parameters need to be selected appropriately. In this section, the grid search method described in the previous work of [57] is employed for automatically setting those tuning parameters of SVM and LSSVM. In addition, the feature selection stage requires the setting of the parameter *r* in the computation of SFs. The model performance with different values of the parameter *r* for the cases of SVM and LSSVM is reported in Figure 12. As can be observed from this figure, *r* = 2 results in the highest CAR_Overall_ values for both SVM and LSSVM.

Besides the two machine learning algorithms of SVM and LSSVM, the backpropagation artificial neural network (BPANN), classification tree (CT), linear discriminant analysis (LDA), and naive Bayes classifier (NBC) are also employed as benchmark classifiers. It is also noted that CT, LDA, and NBC are also equipped with the OvO strategy to deal with the current five-class recognition problem of wall defect classification. The SVM and the benchmark algorithms implemented in MATLAB with the help of the statistics and machine learning toolbox [58]. In addition, the LSSVM model is constructed by the built-in functions provided in the toolbox developed by De Brabanter et al. [59].

In addition, the training phases of BPANN and CT require a proper setting of their model hyper-parameters. Similar to the cases of SVM and LSSVM, the model hyper-parameters of those models that result in the best performance for the testing set are selected. In the case of the DT model, the suitable value of the minimal number of observations per tree leaf is found to be 2. The appropriate structure of the BPANN model consists of 35 neurons in the hidden layers; furthermore, the scaled conjugate gradient algorithm with the maximum number of training epochs = 3000 is used to train the neural network model. Moreover, the processes of selecting an appropriate value of the tuning parameter *r* used in constructing the SF maps of the benchmark models of BPANN, CT, LDA, and NBC are similar to those of the SVM and LSSVM models. Based on result comparisons, the suitable value of the tuning parameter *r* used with BPANN, CT, LDA, and NBC is also 2.

The performances of the machine learning classifiers used for wall damage recognition are summarized in Table 1. Observed from this table, LSSVM has achieved the best predictive performance in terms of CAR_o_ (85.13%), followed by SVM (CAR_o_ = 84.33%), BPANN (CAR_o_ = 74.20%), CT (CAR_o_ = 62.27%), LDA (CAR_o_ = 61.00%), and NBC (CAR_o_ = 59.73%). Thus, it can be seen that classifiers based on LSSVM and SVM are more suitable for the task of wall defect classification than other machine learning and statistical approaches of BPANN, CT, LDA, and NBC.

**Figure 13:**
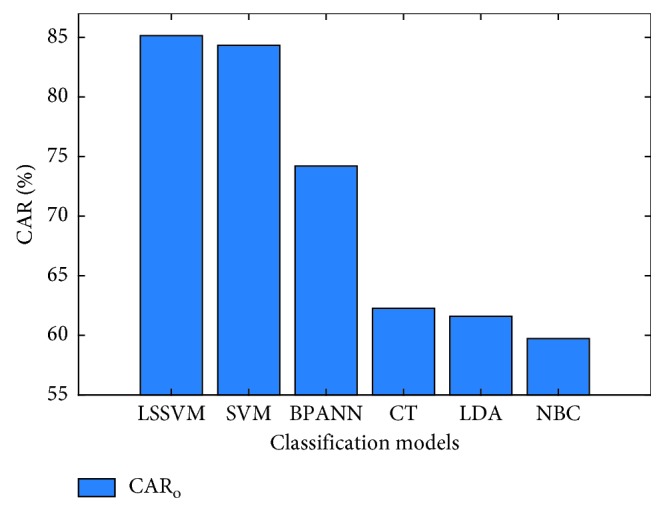
Result comparison based on CAR_o_.

**Figure 14:**
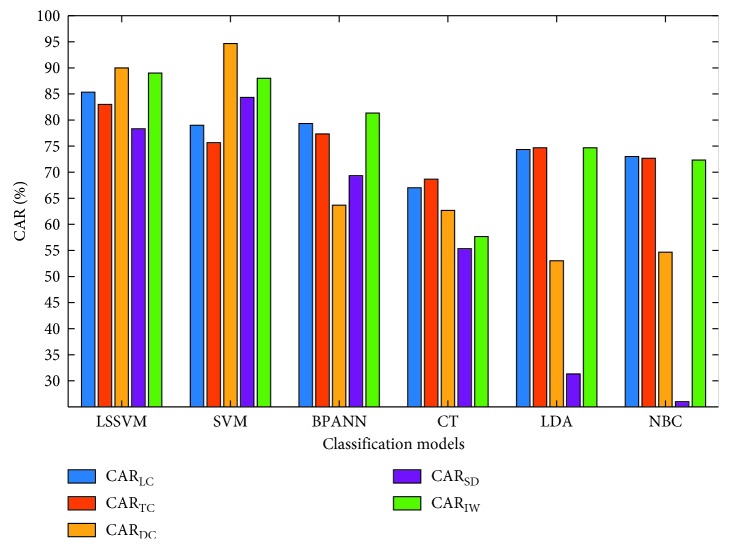
Classification accuracy comparison based on CAR of each class (LD, TC, DC, SD, and IW).

Figures 13 and 14 graphically compare the results obtained from the prediction models in terms of CAR_o_ and CAR of each individual class label. It is shown that the CARs of LC (85.33%), TC (83.00%), and IW (89.00%) obtained from LSSVM are higher than those yielded by SVM (79.00%, 75.67%, and 88.00% for the LC, TC, and IW, respectively). However, results of SVM for the classes of DC (94.67%) and SD (84.33%) are better than those of LSSVM (90.00% and 78.33% for the classes of DC and SD, respectively).

**Figure 15:**
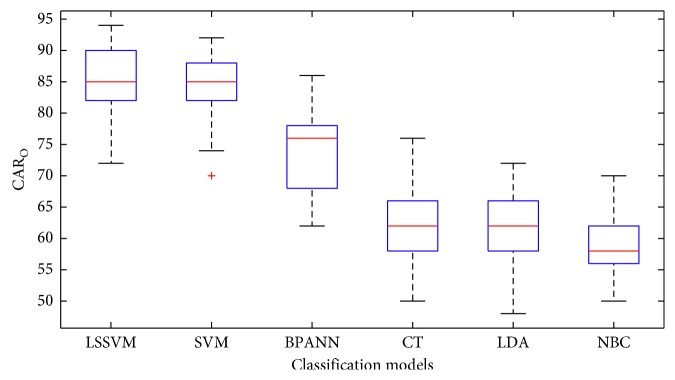
Box plots of overall CARs.

In addition, the classification results of all the models in terms of CAR_o_ are displayed by the box plots in Figure 15. Moreover, to better demonstrate the statistical difference between each pair of classifiers employed in the task of wall defect recognition, the Wilcoxon signed-rank test (WSRT) is utilized in this section. WSRT is a nonparametric statistical hypothesis test that is widely used for verifying the statistical difference of model performances [57]. With the significance level of the test = 0.05, if *p* value computed from the test is smaller than 0.05, it can be confirmed that the performances of the two selected classifiers are statistically different. The *p* values obtained from WSRT for each pair of classifiers are reported in Table 2. The outcomes shown in this table confirm that LSSVM and SVM are significantly better than other benchmark models in the task of recognizing wall damages. In addition, with *p* values = 0.60134, there is no statistical difference between the performances of LSSVM and SVM.

**Figure 16:**
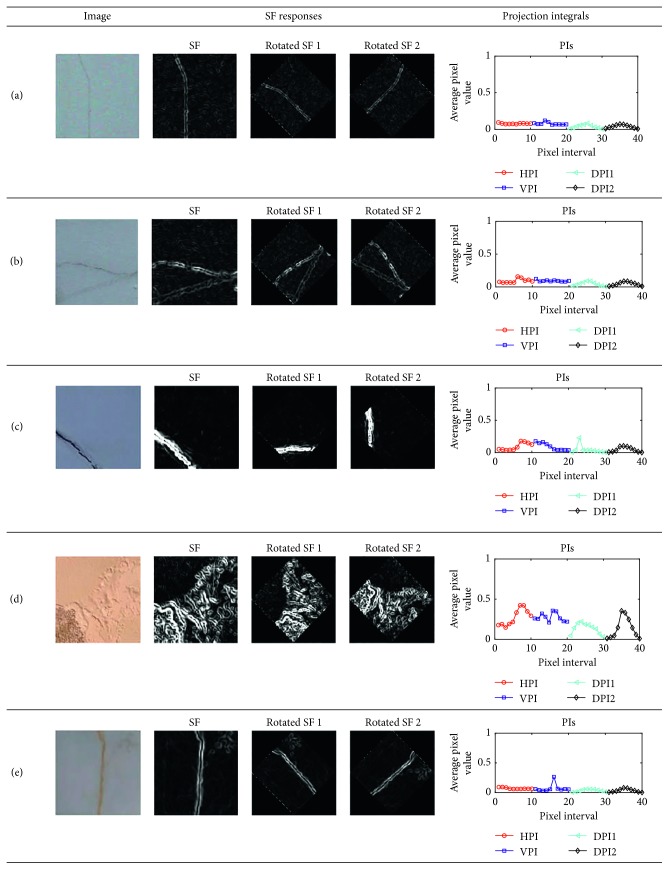
Examples of incorrect classifications.

Although the LSSVM model has delivered the highest CARs, this model also commits wrong classification cases. These misclassifications are investigated and examples of them are illustrated in Figure 16. In Figures 16 and 16, images with the ground truth label of LC and TC have been assigned the label of IW. The reasons for these misclassifications are that the crack objects are too thin; moreover, there is a line of stain existing in Figure 16. The case in Figure 16 shows an image with its ground truth class of DC which has been categorized as the class of TC. The possible reason of this phenomenon is that the crack object appears too close to corner of the image; therefore, the signals of the SF responses of the two DPIs are not significantly stronger than those of other PIs. In Figure 16, an image with complex background texture has caused the machine to misclassify an image with the ground truth category of SD. Moreover, an object of stain (Figure 16) leads to the classification of an image into the class of LC while it actually belongs to the class of IW.

## 6. Conclusion

This study has proposed an automatic approach for periodic survey of concrete wall structures. The newly constructed approach mainly consists of the feature extraction step and the pattern classification step. In the first step, image processing techniques of SF and PI have been employed to characterize the texture and the pattern existing in images. In the second step, machine learning algorithms of SVM and LSSVM have been used to analyze the features extracted by the image processing techniques and to assign input images into one of the five class labels of LC, TC, DC, SD, and IW. Experimental results using a repeated random subsampling with 20 runs show that the predictive performances of LSSVM (CAR_o_ = 85.13%) and SVM (CAR_o_ = 84.33%) are superior to other benchmark models of BPANN, CT, LDA, and NBC. These facts confirm that the proposed approach can be a time- and cost-effective solution for the task of building periodic survey. The future developments of the current study include the integration of other advanced image processing methods (e.g., image segmentation, color/texture analyses) to enhance the CARs via the reduction of falsely classified cases.

## Figures and Tables

**Table 1:** Result comparison.

Statistics	CAR (%)	Classification models					
		LSSVM	SVM	BPANN	CT	LDA	NBC
Average	CAR_LC_	85.33	79.00	79.33	67.00	74.33	73.00
	CAR_TC_	83.00	75.67	77.33	68.67	74.67	72.67
	CAR_DC_	90.00	94.67	63.67	62.67	53.00	54.67
	CAR_SD_	78.33	84.33	69.33	55.33	31.33	26.00
	CAR_IW_	89.00	88.00	81.33	57.67	74.67	72.33
	CAR_O_	85.13	84.33	74.20	62.27	61.60	59.73

Std.	CAR_LC_	10.42	12.69	11.12	16.01	17.94	13.43
	CAR_TC_	10.22	15.24	17.21	13.83	12.24	11.43
	CAR_DC_	11.74	7.30	14.26	13.11	16.43	17.56
	CAR_SD_	13.41	11.35	17.80	16.97	15.02	12.76
	CAR_IW_	10.62	8.05	13.58	18.88	12.79	13.05
	CAR_O_	5.89	5.28	6.75	5.80	6.53	5.63

**Table 2:** *p* values of the Wilcoxon signed-rank test.

	LSSVM	SVM	BPANN	CT	LDA	NBC
LSSVM	0.00000	0.60134	0.00001	0.00000	0.00000	0.00000
SVM	0.60134	0.00000	0.00002	0.00000	0.00000	0.00000
BPANN	0.00001	0.00002	0.00000	0.00000	0.00000	0.00000
CT	0.00000	0.00000	0.00000	0.00000	0.06728	0.00444
LDA	0.00000	0.00000	0.00000	0.06728	0.00000	0.02230
NBC	0.00000	0.00000	0.00000	0.00444	0.02230	0.00000

## Data Availability

The data used to support the findings of this study are available from the corresponding author upon request.

## Supplementary Materials

Supplementary Materials The supplementary file contains the data set used in this study. In this file, the first 500 columns are the input features (X1 to X40) of the data (which are the smoothed projection integrals); the last column is the class labels (1 = longitudinal crack, 2 = transverse crack, 3 = diagonal crack, 4 = spall damage, and 5 = intact wall).
